# Beyond the Pain: Rethinking Chronic Pain Management Through Integrated Therapeutic Approaches—A Systematic Review

**DOI:** 10.3390/ijms27031231

**Published:** 2026-01-26

**Authors:** Nicole Quodling, Norman Hoffman, Frederick Robert Carrick, Monèm Jemni

**Affiliations:** 1 The Carrick Institute, Cape Canaveral, FL 32920, USA; drnorm@hoffmanwellness.com (N.H.); drfrcarrick@post.harvard.edu (F.R.C.); monemj@hotmail.com (M.J.); 2 Coelevate Chiropractic, Adelaide, SA 5081, Australia; 3 Burnett School of Biomedical Sciences, University of Central Florida, Orlando, FL 32827, USA; 4 MGH Institute of Health Professions, Boston, MA 02129, USA; 5 Centre for Mental Health Research in Association with the University of Cambridge, Cambridge CB3 9AJ, UK; 6 College of Medicine, University of Central Florida, Orlando, FL 32827, USA; 7 Faculty of Physical Education, Ningbo University, Ningbo 315211, China

**Keywords:** central sensitization, fibromyalgia, complex regional pain syndrome, neuropathic pain, vision, audition, olfaction, touch, taste, proprioception

## Abstract

Chronic pain is inherently multifactorial, with biological, psychological, and social factors contributing to neuropathic pain (NP) and central sensitization (CS) syndromes. Comorbidity between functional disorders and the lack of clinical biomarkers adds to the challenge of diagnosis and treatment, leading to frustration for healthcare professionals and patients. Available treatments are limited, increasing patient suffering with personal and financial costs. This systematic review examined multisensory processing alterations in chronic pain and reviewed current pharmacological and non-pharmacological interventions. A structured search was conducted on the PubMed database using the keywords Central Sensitization, Fibromyalgia, Complex Regional Pain Syndrome, and Neuropathic Pain, combined with the keywords Vision, Audition, Olfaction, Touch, Taste, and Proprioception. Papers were then filtered to discuss current treatment approaches. Articles within the last five years, from 2018 to 2023, have been included. Papers were excluded if they were animal studies; investigated tissue damage, disease processes, or addiction; or were conference proceedings or non-English. Results were summarized in table form to allow synthesis of evidence. As this study is a systematic review of previously published research rather than a clinical trial or experimental investigation, the risk of bias was assessed independently by at least two reviewers. 138 studies were identified and analyzed. Of these, 96 focused primarily on treatment options for chronic pain and were analyzed for this systematic review. There were a few emerging themes. No one therapy is effective, so a multidisciplinary approach to diagnosis, including pharmacological, somatic, and psychological treatment, is generally predicted to achieve the best outcomes. Cranial neurovascular compromise, especially of the trigeminal, glossopharyngeal, and potentially the vestibulocochlear nerve, is being increasingly revealed with the advancement of neuroimaging. Cortical and deep brain stimulation to evoke neuroplasticity is an emerging and promising therapy and warrants further investigation. Finally, including patients in their treatment plan allows them control and offers the ability to self-manage their pain. Risk of bias limits the ability to judge the quality of evidence.

## 1. Introduction

Pain is an unpleasant sensory and emotional experience [1]. Its purpose is to protect and warn about potential tissue damage signaled by receptors and fiber systems from the peripheral nervous system to the cortex. Centralized pain is theorized to be a dysfunction of the nervous system rather than an adaptive change, so as well as being not protective it is also maladaptive [2,3]. Patients commonly present with persistent physical pain symptoms for which no identifiable disease cause can be established. This presentation is prevalent across all medical disciplines and can be challenging for both patients and practitioners, particularly when symptoms are distressing and not adequately explained by conventional diagnostic frameworks. Chronic pain is inherently multifactorial, with biological, psychological, and social factors contributing to neuropathic pain (NP) and central sensitization (CS) syndromes [4,5]. Although various etiologies can cause nociplastic pain, the symptoms and characteristics of pain are influenced by pathophysiological mechanisms rather than the etiology, with considerable comorbidity between functional disorders, with important therapeutic implications for the personalized treatment of NP [6,7,8]. Traditionally, physicians attributed pain without a physical cause as psychological or a diagnosis of exclusion [4]. People living with chronic pain without overt tissue damage often feel invalidated, as, despite significant evidence, debate regarding neuropathic and centralized pain still occurs [4,9]. However, pain without a definable biological process is still genuine [4]. The global economic burden of chronic pain is substantial, associated with a reduction in productivity and quality of life (QoL) and increased costs to the health system. Lack of awareness regarding chronic pain can negatively impact the time to accurate diagnosis and the level of care provided [9]. Available treatments are limited [10], increasing patient suffering with personal and financial costs [9]. Pain management, regardless of its origin, is twofold: to identify the origin of the pain and provide relief [4]. There is a need for collaborative therapies that can modulate the experience of pain and enable a better QoL for our patients [10]. This systematic review focused on the association of chronic pain syndromes with changes in sensation and then distilled current and emerging treatment protocols from the literature. Therefore, the aims of this systematic review were to explore alterations in multisensory processing associated with chronic pain conditions and to review current pharmacological and nonpharmacological treatment strategies. The findings underscore the heterogeneity of treatment responses and support a multidisciplinary approach to chronic pain management.

## 2. Methods

**Figure 1:**
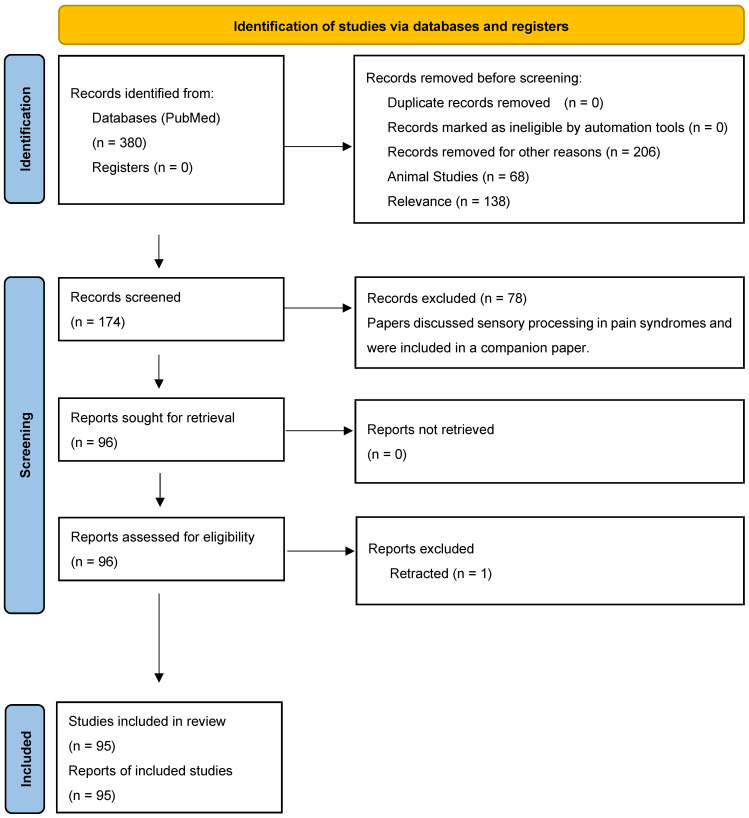
PRISMA framework [11].

Registration of this review was not performed; however, the review was conducted in accordance with PRISMA guidelines (Supplementary Materials) [11]. Following the PRISMA framework (Figure 1), a structured search was conducted on the PubMed database using the keywords Central Sensitization, Fibromyalgia, Complex Regional Pain Syndrome, and Neuropathic Pain, combined with the keywords Vision, Audition, Olfaction, Touch, Taste, and Proprioception. Papers were then filtered to discuss current treatment approaches. PubMed was chosen because it is accessible and user friendly. Articles within the five-year time frame, from 2018 to 2023, were included. Papers were excluded if they were animal studies; investigated tissue damage, disease processes, or addiction; or were conference proceedings or non-English. Previous reviews were included to summarize evidence from different outcomes, conditions, or populations. Key characteristics of each study were extracted and summarized in table form to enable efficient narrative synthesis. As this study is a systematic review of previously published research rather than a clinical trial or experimental investigation, the risk of bias was assessed independently by at least two reviewers.

## 3. Results and Discussion

380 studies on conditions of CS and sensory processing were identified. After title and abstract screening, 174 studies were identified as meeting inclusion. These papers were then sorted into two categories—those primarily discussing sensory processing in pain syndromes and those discussing treatment options. A total of 96 papers predominantly discussed treatment options for nociplastic pain and were included in this paper. One paper was subsequently retracted and removed. The remaining 78 papers predominantly discussed sensory processing in nociplastic pain and were included in a companion paper. Interventions and observations are summarized in Table 1.

### 3.1. Neuropathic Pain (NP)

Neuropathic pain (NP) is caused by maladaptive neuroplastic responses that follow damage to the nervous system [2,12,13,14,15] and persist for at least three months or beyond the expected time for healing [6]. A variety of conditions can initiate NP, including peripheral nerve injury and central nervous system (CNS) injury [1,2,16,17], viral infection, tumors [18,19], and metabolic disorders [17]. In contrast to nociceptive pain, which is adaptive and detected in response to potentially tissue damaging noxious stimuli, NP is caused by a functional disturbance of the neuroaxis that is not due to tissue damage or peripheral alterations, characterized by spontaneous pain and symptoms of sensory loss or sensory gain associated with allodynia or hyperalgesia [5,6,13,17,20,21]. Descending pathways modulate spinal cord output through neurotransmitters, including noradrenaline, serotonin (5-HT), gamma-aminobutyric acid (GABA), and endogenous opioids [2,15]. Noradrenergic signaling mediated via α2-adrenoceptors plays a predominantly antinociceptive role in pain modulation, whereas serotonergic effects on nociception are receptor subtype dependent, with both pronociceptive and antinociceptive effects [2]. Evidence suggests that, under physiological conditions, descending noradrenergic and serotonergic inhibitory pathways predominate over serotonergic facilitatory signaling; however, this balance may shift during chronic pain [1]. Concurrently, upregulation of proexcitatory ion channels enhances neurotransmitter release, excitability, and ectopic firing of sensory neurons [1,4]. NP syndromes are particularly refractory to treatment and impose substantial suffering [1,14,17,22,23]. The estimated prevalence of NP has varied from 0.9% to 20% [12,15,17,20,21,23,24,25,26]. Females have a higher incidence of comorbid conditions [27]. Individuals diagnosed with NP are among the most frequent consumers of healthcare services, generating high costs both at the individual and societal levels [14,23,25]. Since there are no pain biomarkers, NP is identified based on clinical criteria. However, the physical examination can only provide supporting evidence for a neurological lesion or disorder that could genuinely be the cause of pain [15]. Despite recent advances in understanding, diagnosing, and treating NP, effective treatments remain to be discovered. Most patients do not experience complete pain relief with currently available treatments [17,23,26].

### 3.2. Complex Regional Pain Syndrome (CRPS)

Complex regional pain syndrome (CRPS) is an example of a NP syndrome. It is characterized by pain arising in one or more limbs, temporally and spatially disproportionate to the inciting event and associated with trophic changes and sensory, motor, and autonomic dysfunction [4,9,28,29,30,31,32,33,34]. CRPS usually develops from a peripheral event, but its maintenance relies on changes within the CNS [9,28,30,31,32,35], associated with inflammation, autoimmunity, genetics, dysregulation of the sympathetic nervous system, the accumulation of nociceptive neurotransmitters and maladaptive neuroplasticity [28,29,30,31,32,35]. Evidence indicates that noradrenaline contributes to the pathophysiology of CRPS through altered sympatho-nociceptive coupling. Preclinical post ischaemic injury models reported increased nociceptor firing following noradrenaline exposure, alongside upregulation of adrenergic receptors on nociceptive fibers after nerve trauma, allowing direct sympathetic activation [31].

Clinical diagnosis can be challenging as CRPS follows a regional pain distribution rather than a dermatomal or peripheral nerve pattern [30]. These changes may be responsible for diminished perception associated with changes in multisensory integration and somatosensory representation in the brain [9,31,35]. The estimated incidence of CRPS is 5.5–26.2 cases per 100,000 people per year [28,29,34], with females affected more than males [9,34]. The pathogenesis of CRPS remains obscure and limited, leading to delayed diagnosis or misdiagnosis [9,30,32,34]. The debilitating consequences of CRPS contribute to a significant reduction in the QoL and an increased risk of suicide compared to people with other chronic pain conditions [9,30]. Physical impairment and severe pain continue two years after the initial onset for approximately 15% of diagnosed patients [32,33], and 31% are not back to work 2 years after the onset of symptoms [32]. Osteopenia and patchy osteoporosis, which can be detected on three phase bone scans, can be seen in the early stages. Sweat testing, diagnostic sympathetic blocks, and quantitative sensory testing can be helpful in evaluating CRPS. However, there are no definitive diagnostic tools [28] and almost no evidence to support therapies currently used in CRPS [30,32].

### 3.3. Central Sensitization (CS)

Central sensitization (CS) occurs with heightened excitation of the somatosensory nervous system and dysfunction of endogenous pain inhibition with resultant amplification of neural signaling in response to mechanical or other sensory stimuli, including light, sound, and temperature [21,27,36,37]. Studies reported that CS was associated with neuroimmune and neurotransmitter alterations, including microglial release of proinflammatory cytokines and brain-derived neurotrophic factor (BDNF), leading to a shift in GABAergic and glycinergic signaling in spinal lamina I from inhibitory to excitatory, facilitating activation of dorsal horn nociceptive neurons. Upregulation of 5-HT3 receptors in dorsal horn laminae I–II was associated with reduced noradrenergic inhibitory modulation [2,17]. Additionally, descending modulation mediated by endogenous opioids from the rostroventromedial medulla was described as activating antinociceptive cells and inhibiting pronociceptive cells projecting to the spinal dorsal horn [2,3]. CS may occur after persistent acute peripheral nerve injury and is inferred indirectly from allodynia or hyperalgesia [2,7]. Centralized pain is associated with mood changes, fatigue, cognitive disturbances, sleep changes, catastrophizing, and often comorbid major depressive disorder or generalized anxiety disorder [5]. Psychological stressors can also trigger worsening symptoms with a strong association with maladaptive psychosocial factors, including negative emotions, poor self efficacy, maladaptive beliefs, and pain behaviors [2,37]. Synaptic plasticity, underlying learning and memory, is a particular component of CS [8,32], so early life trauma or emotional stress can contribute to centralized pain in patients [38]. There may be a genetic component to widespread pain [29,38]. Centralized pain occurs in 6–10% of the general population, most of whom have fibromyalgia (FM) [12]. Central pain syndrome can be a disabling illness that causes significant deterioration to a patient’s QoL and is resistant to current known etiologic treatments, often with modest or conflicting results [13,39].

### 3.4. Fibromyalgia (FM)

Fibromyalgia (FM) is a complex multifactorial condition of unknown etiology characterized by chronic widespread pain, hyperalgesia, and allodynia [17,38,39,40,41,42], leading to significant disability [36,38]. Patients present with multiple sites of pain or tender points, fatigue, cognitive impairment, sleep impairment, and emotional or mood fluctuations [12,17,39,40,41,42,43,44,45]. Individuals with FM syndrome may experience proprioceptive and balance impairments, gait alteration, sensorimotor deficits, and distortions of body representation, contributing to a greater prevalence of falls [38,39,42,44]. Risk factors for FM parallel the risk factors for CS, including stress, obesity, and family history; however, the literature is not conclusive, and it is still considered a condition of unknown cause [8,17,20,39]. Dysfunction of the central, autonomic, and peripheral nervous systems, alterations of neurotransmitters, endocrine, immune, and inflammatory systems, oxidative stress, external stressors, and psychological factors leading to amplified sensory processing have been implicated [17,38]. Signs and symptoms of dysautonomia have been observed in patients with FM, characterized by persistent autonomic nervous system hyperactivity at rest and hyporeactivity during stressful situations, which may explain some of the multisystem features [41]. The total prevalence of FM in the general population ranges from 0.2% to 11% and is most frequent in women [4,12,20,36,38,44,46,47]. Chronic pain and fatigue associated with FM significantly impair activities of daily living and reduce QoL [40]. For many patients, these symptoms persist for years, leading to frequent healthcare use [39]. Because a pattern of pathological, imaging, or biochemical features has not yet been characterized, the etiopathogenesis of FM is still uncertain, and there is no specific effective therapy [17,39].

### 3.5. What Are the Current Pharmacological and Surgical Treatments?

Pharmacological and interventional therapies for symptomatic relief are the mainstay of treating medically unexplained pain [14,19]. Conventional treatments include medication, rehabilitation and, ideally, psychological therapy [32]. Pharmacotherapy remains the principal treatment modality, although the efficacy of individual drugs is variable [44]. Traditional pain relievers, such as nonsteroidal antiinflammatories (NSAIDs) or opioids, often are not helpful [4], and antidepressants and anticonvulsants are prescribed as an adjunct or alternative [21,23,47,48]. Antidepressants have the added benefit of treating comorbid mood disorders [23]. However, there is only a limited number of drugs with proven efficacy in the treatment of nociplastic pain, and most are associated with considerable adverse side effects [24,49]. Many patients cannot obtain sufficient pain relief from medication alone, and interventional strategies, such as nerve blocks and neuromodulation, are used as indicated [26,30]. Essential adjunctive treatment strategies are patient education for self-management and the regular inclusion of psychological intervention [28].

#### 3.5.1. Antiinflammatory Medications

Antiinflammatory medications are a first-line treatment for pain [6,27,28,29,34,50,51,52]. NSAIDs inhibit inflammatory cyclooxygenase-1 (COX-1) and inflammatory cyclooxygenase-2 (COX-2), reducing prostaglandins and pain perception [2]. However, NSAIDs often cause a variety of adverse effects with prolonged use, including upper gastrointestinal disease, cardiovascular issues, renal and liver failure, and death in high doses [2,17,51,53,54]. Evaluation of the daily use of 1000 mg of naproxen and diclofenac (dose not specified) reported consistent findings across subgroup analyses of age, gender, race, and ethnicity [52]. The use of NSAIDs is suggested if there is good evidence of improved efficacy over paracetamol [2,43].

#### 3.5.2. Analgesics

Several reviews and international guidelines recommend paracetamol as the first-line analgesic to treat chronic pain [2,43,52]. Paracetamol was reported to provide analgesia primarily through inhibition of cyclooxygenase enzymes, particularly COX-2, resulting in reduced prostaglandin synthesis. Paracetamol is in first-line use in chronic pain due to its favorable safety profile, efficacy, low cost, and reduced reliance on NSAIDs [2]. Weekly dexmedetomidine 200 µg intravenous sedation has been prescribed for pain control in CRPS [28]. Systemic analgesics show mixed results for NP [21,23,53] and FM [27,44]. There were no subgroup analyses for age, gender, race, or ethnicity [52]. Paracetamol is considered safe, but high doses are associated with liver disease and death [2,52].

#### 3.5.3. Selective Serotonin and Serotonin-Noradrenaline Reuptake Inhibitors (SSRI/SNRI)

Selective serotonin and serotonin-noradrenaline reuptake inhibitors (SSRI/SNRI) are used as second-line agents or as first-line agents in certain patients, such as those with concomitant depression [6,8,20,21,28,44,50]. Antidepressants have the added benefit of treating comorbid mood disorders [21,23]. In the short term, minor improvements in pain and function are seen with selective noradrenaline reuptake inhibitor (SNRI) use for NP and FM [49,52,54], but they do not provide any clinically relevant benefit over placebo in improving health related QoL, reducing fatigue, or reducing sleep problems [55]. The effectiveness of duloxetine in pain control has not been found to differ among gender and race subgroups [52]. Antidepressants can disturb balance and are related to falls [43]. Other common adverse effects include headaches, blurred vision, nausea, drowsiness, sleep disturbances, and hyperhidrosis [2,49]. Dose reductions reduce the risk of some adverse events [54], but evidence regarding the effectiveness of antidepressants for chronic pain conditions is conflicting [2,8,26,44].

#### 3.5.4. Triptans

Triptans, the agonists of 5-HT 1B/D receptors, are among the most effective drugs for severe acute migraine attacks [52,53]. Triptan responders have clinical and biochemical evidence of increased trigeminal activation [56]. They can, however, induce adverse effects, including chest, face, and limb pain that is difficult to tolerate by many patients. Moreover, they are vasoconstrictors and are contraindicated in patients with comorbid cardiac or cerebrovascular pathology [52,53].

#### 3.5.5. Anticonvulsants

Anticonvulsants such as carbamazepine and oxcarbazepine are prescribed for neuropathic and centralized pain, CRPS and FM; however, results are conflicting [16,19,20,27,28,30,34,37,44,48,52,53,54,56,57,58,59,60,61,62]. Minor improvements in pain and function have been reported with oxcarbazepine for NP [54], although its efficacy has been primarily limited to trigeminal neuralgia (TN) [63,64]. Second-line agents include the anticonvulsants lamotrigine and topiramate for TN [63,64] and migraine [53,65]. They can be used either as an addition to or as monotherapy should the first-line medication not be sufficient [57,58]. However, long-term treatment with anticonvulsants has been associated with many side effects, including sleepiness, tiredness, dizziness, nausea, vomiting, impaired motor function, renal and hepatic toxicity, allergic reactions [16,57,63,66], diplopia, and photosensitivity reactions, especially in females [65], and their efficacy decreases over time [2,16,53,63,67]. Evidence supporting the use of anticonvulsants for chronic pain is scarce [1,8,67].

#### 3.5.6. Monoclonal Antibodies

Research suggests that Calcitonin Gene Related Peptide (CGRP) released at the central terminals of nociceptors facilitates CS [1,56]. Monoclonal antibodies targeting CGRP or its receptor prevent episodic and chronic migraines [56]. A prospective study on 864 migraine patients who received subcutaneous erenumab (70 mg or 140 mg, monthly), galcanezumab (120 mg monthly, following a 240 mg loading dose), or fremanezumab (225 mg, monthly or 675 mg, quarterly) found the strongest predictor of response was most associated with headache features indicative of trigeminal activation, being unilateral pain, unilateral autonomic symptoms, and allodynia. These findings support the value of pain profiling to guide more personalized use of anti-CGRP therapies [56]. There is an excellent efficacy and tolerability ratio, representing a substantial step forward in the care of migraine patients [53,56]. The effectiveness of monoclonal antibodies in treating migraine supports the role of CGRP in migraine pathophysiology [50,56]. Obesity can contribute to decreased efficacy, so weight reduction strategies could be advantageous in increasing anti-CGRP monoclonal antibody responsiveness [56].

#### 3.5.7. Gabapentinoids

Pregabalin and gabapentin are similar compounds with analgesic, anticonvulsant, and anxiolytic characteristics [68] and are used in isolation or in conjunction with first-line medication [12,57]. Gabapentinoids have been approved for peripheral NP and FM [12,13,20,26,27,28,29,30,44,52,61,62,68,69] and are one of the main treatment options for patients with CRPS [21,28,30]. Gabapentinoids reduce voltage-gated calcium channel currents in the CNS, thereby reducing the release of stimulatory neurotransmitters, increasing neuronal GABA levels, and diminishing excitation or increasing inhibition of pain pathways [2,6,8,44,50,68,70]. The initial and maximum daily doses prescribed for males tend to be higher than those prescribed for females, but with similar prescription periods [12]. The prescription of gabapentinoids in the treatment of CS and NP [54] is debated [52]. Pregabalin is initiated at 150 mg/day but demonstrates inconsistent efficacy at this dose. Prescribed doses frequently remain below the recommended maintenance range (≥300 mg/day), suggesting that subtherapeutic dosing may contribute to variable clinical effectiveness [12]. Adverse effects include swelling, confusion, dizziness, headache, nausea, peripheral edema, and diplopia [2,54,68,70]. Many patients cannot obtain sufficient pain relief or do not tolerate adequate doses of systemic drug therapies because of side effects [23,26].

#### 3.5.8. Tricyclic Antidepressants

Tricyclic antidepressants (TCA) show conflicting evidence for neurogenic pain [8,19,20,26,27,30,34,50,52,53,67] and CRPS [30,34]. TCAs can be efficacious in NP [2,19,20,21]. Their efficacy in decreasing pain sensitivity is mediated by inhibiting 5-HT and noradrenaline reuptake in the descending pain modulatory pathways [2,8]. TCAs may cause adverse effects, including orthostatic hypotension, dry mouth, urinary retention, constipation, and cardiotoxicity and are associated with modest improvement [2,8,54].

#### 3.5.9. Glucocorticoids, Immunoglobulins, Bisphosphonates, and Muscle Relaxants

Glucocorticoids [28,30,34], intravenous immunoglobulin [4], and bisphosphonates have been used for the treatment of CRPS [28,34], but results have been inconsistent [4]. Drug-induced myopia has also been associated with corticosteroids [65]. Centrally acting muscle relaxants have been employed to manage CS through the inhibition of noradrenaline uptake, reducing hypertonus [8], and as a second-line agent for TN [48,58,63]. Muscle relaxants can impair balance and have been associated with falls [43].

#### 3.5.10. Opioids

Across the included studies, opioid use was reported to be associated with a range of adverse effects, including the development or worsening of NP and allodynia [20,24]. Opioid therapy was primarily reported for short-term use [2,4], as long-term exposure was associated with effects on reward pathways, increased risk of dependence, and reduced analgesic benefit over time due to the development of tolerance [5,23,27,51,71]. Chronic use of conventional opioids was reported to be associated with opioid-induced hyperalgesia [5,20,21], attributed to sustained activation of μ-opioid receptors [2]. Modulatory effects of genetic variants in genes encoding the μ-opioid receptor were also reported to influence pain processing and opioid responsiveness [24]. Collectively, these findings were associated with limited efficacy of conventional opioids in chronic pain and NP states, increased adverse effects, and heightened pain sensitivity, supporting reported limitations of opioid therapy in chronic musculoskeletal pain [2,24].

In addition, several studies reported that opioids were frequently perceived as effective by patients with chronic NP despite limited evidence of benefit [20]. Guidelines referenced in the included studies classified opioids as third-line agents for NP, reflecting concerns regarding limited efficacy, risk of dependence, and potential for worsening long term pain outcomes [20]. High rates of opioid misuse and dependence were also reported, underscoring the need for alternative approaches to chronic pain management [72].

#### 3.5.11. Cannabinoids

There has been hope for cannabinoids as a treatment for NP [10,70,71,73]; however, data are still limited [8,15,20,52,70]. The endocannabinoid system is an important modulator of the stress response, helping restore equilibrium after a sympathetic nervous system event, and has potential analgesic effects [10,15,71]. Cannabinoid receptors activate potassium channels and inhibit voltage-gated sodium channels, inhibiting the release of neurotransmitters at the synapse [10]. Cannabinoid type 1 (CB1) receptors are predominantly found in the frontal cortex, basal ganglia, and cerebellum, and to a lesser extent in chondrocytes and osteocytes, resulting in primary CNS effects. Cannabinoid type 2 (CB2) receptors are predominantly found on immune cells, chondrocytes, and osteocytes, resulting in primary immunological effects. CB1 receptors modulate immune cells and inhibit the release of inflammatory mediators, with potential analgesic effects [10,70]. Transient receptor potential (TRP) channels are membrane proteins that transduce many chemical and physical stimuli, with dysfunction implicated in NP. In addition to CB1 and CB2 receptors, cannabinoids can modulate TRP vanilloid (TRPV), TRP ankyrin (TRPA), and TRP melastatin (TRPM) subfamilies, providing a promising target for the treatment of pain [71]. TRPV1 is a ligand-activated cation channel, regarded mainly as a pain receptor. It is expressed on the C-fiber and Aδ sensory neurons. Cannabinoid activation can cause a reduction in TRPV1 activity, leading to reduced interleukin-6 (IL-6) secretion [10], although in animal studies these effects seem to be less prominent in female subjects [70]. The benefits of exogenous cannabinoids include analgesic, antiinflammatory, antiemetic, and anticonvulsive effects and improved mood state, cognition, and appetite, providing promising results in the treatment of chronic pain conditions [15,70]. However, common adverse effects of cannabinoids include dizziness, nausea [55], dry mouth, tachycardia, and negative psychological impact [10,15]. Cannabinoid induced drug–drug interactions include diarrhea, vomiting, fatigue, somnolence, and hepatic abnormalities [73]. Concomitant use of cannabinoids with gabapentin and pregabalin may cause additive sedative effects. Caution has been recommended for elderly patients, those with unstable mental health disease, or those receiving concomitant therapy with psychoactive drugs [10]. Benefits of cannabinoid use for pain syndromes have been empirically reported [10,15,70]; however, research shows inconsistent effects on pain [55], and they are not recommended due to CNS-mediated adverse affects [15].

#### 3.5.12. N-Methyl-D-aspartate Receptor (NMDAR) Antagonists

Ketamine is a dissociative pain medication that exerts its action by inhibiting the N-methyl-d-aspartate receptor (NMDAR), exerting muscarinic and opioid effects, and has been used in the treatment of CRPS [4,28,34], NP [67], and FM [52]. Ketamine has failed to produce significant functional improvement; however, there is reported improvement in VAS pain scales [4], and some studies have shown long-term effects of ketamine for CRPS, possibly through desensitization of NMDAR in the CNS [28]. However, NMDAR agonists are not always well tolerated by patients [67] and have psychomimetic side effects, such as hallucination, which limits their use [28].

#### 3.5.13. Botulinum Toxin

Botulinum toxin primarily acts by preventing acetylcholine release at the neuromuscular junction, thereby blocking neurotransmission [58,74], as well as by reducing synaptic release of CGRP [50]. The injection of botulinum toxin into trigger zones in TN [48,58,59,63,66], peripheral NP [1,6,20,21,66] and migraine [50,56] provides rapid pain relief with minimal side effects [20,34,59,74] and may provide a promising alternative to surgery for individuals whose pain is unresponsive to medication [34,48,56,58]. Botulinum toxin targets pain responses by reducing muscle contraction [20,74] and thus decreasing afferent signaling [50]. Repeated injections have a high economic cost and provide only short-term symptomatic relief [59,66].

#### 3.5.14. Nerve Blocks

Across the included studies, peripheral nerve blocks using local anesthetics were less frequently reported interventions for pain localized to specific neuropathic distributions and were described as having both diagnostic and therapeutic roles [1,4,17,21,26,28,30,34,50,59,66,75]. Local anesthetics, most commonly lidocaine or bupivacaine and often combined with corticosteroids, were administered to block injured nerve afferents, typically via perineural injection under ultrasound guidance [17,75]. The most commonly reported targets were the greater occipital nerve for headache, refractory ocular pain, and migraine [50,75]; the trigeminal and intercostal nerves for chronic NP [19,20,63]; and the sphenopalatine and stellate ganglia for migraine, dry eye, and CRPS [6,28,30,34,50]. Symptom relief was reported to occur within minutes, with variable duration ranging from hours to several months, and repeat injections were described for recurrent pain, commonly at three-month intervals [75]. Injection of local anesthetic into pelvic floor muscles was also reported to interrupt pelvic pain pathways resulting from endometriosis through muscle relaxation and lengthening [8].

Across conditions, nerve blocks were reported to provide benefit in selected patients with refractory pain [50]; however, limited duration of effect [20,75], reduced efficacy in centrally mediated pain states due to concurrent CNS dysregulation [6], lack of improvement in non-pain CRPS symptoms [4], and risks including bleeding, infection, neuritis, and pain exacerbation were also reported [75].

#### 3.5.15. Spinal Stimulation

Spinal cord stimulation uses pulsed electrical energy near the spinal cord to manage pain [34,76]. One study reported that Burst dorsal root spinal cord stimulation (500 Hz) was associated with normalization of tactile detection and Aδ fiber measures, and tonic spinal cord stimulation (50 Hz) was associated with reduced cold pain tolerance [77]. It is hypothesized that by altering local neurochemistry, stimulation suppresses the hyperexcitability of the wide dynamic range of neurons by increasing GABA and 5-HT release and suppressing levels of the excitatory cytokines glutamate and aspartate. Pain impulses provoked in the periphery, carried by C and A-delta fibers, could be interrupted by stimulating larger A-beta fibers. This interruption is facilitated by the common nerve synapse location in the substantia gelatinosa of the dorsal horn [76]. This modality can benefit patients with many types of neuropathic and radicular pain refractory to other procedural interventions [20,43,76,77]. However, spinal cord stimulation is invasive, requiring minor surgery and implantation of an electrical device [4,20], requiring follow up, reprogramming, wound management, and wireless recharging. Ultimately, spinal cord stimulation requires the patient’s active participation in their care and the responsibility of continuously managing their pain [76]. Complications range from infection to inappropriate paresthesia coverage, lead migration or breakage, hematoma, nerve injury, paralysis, and death [76]. Most studies regarding spinal cord stimulation therapy are either small prospective studies or retrospective studies. For this reason, there are relatively few guidelines regarding contraindications [21,76].

#### 3.5.16. Microvascular Decompression (MVD)

Neurovascular compression occurs when vessels contact a cranial nerve, resulting in abnormal sensory or motor symptoms [66,78]. The most common manifestations are TN [10,57,66,79] and hemifacial spasm symptoms [66,78]. TN is classically caused by neurovascular compression [63,64,69], most frequently by the superior cerebellar artery [60,63,80]. Other vessels responsible for trigeminal compression include the anterior inferior cerebellar artery, basilar artery, and pontine veins [63,80]. However, TN may be related to other etiologies, thus presenting different and additional features [57,69,78]. Microscopic microvascular decompression (MVD) of the trigeminal nerve is best practice surgical treatment for medically refractory classical TN [19,48,54,57,58,60,63,64,69,79,80,81,82,83,84]. Due to the success of MVD in the treatment of TN, vascular compression has been speculated to be the etiology of other cranial neuropathies associated with significant symptomatology as a function of the involved cranial nerve [78]. Other indications for MVD surgery include glossopharyngeal neuralgia [60,62,66,79,80,82,83,84], occipital neuralgia, tinnitus [66,78], and vagal palsy [66,78,83]. MVD is effective with clinically significant outcomes [57,83]. However, complications are relatively frequent [57,82] and include infection, facial palsy, facial numbness, cerebrospinal fluid leak, and hearing reduction or loss [22,54,57,63,64,79,84,85]. While there is a risk of surgical complications after MVD, this needs to be weighed against the excruciating and intense pain, rendering the patient severely affected [57,63]. Radiofrequency ablation [19,54,61,62,66], rhizotomy [61,62,85,86], and percutaneous balloon compression [19,66,69,86] have emerged as promising alternatives but are not free from complications, including hypoesthesia, masticatory muscle weakness, visual disturbance, temporal muscle atrophy, facial hematoma, and reactions to anesthesia [29,54,86].

#### 3.5.17. Topical Medical Pain Management

Topical medications may be helpful for allodynia, including lidocaine [13,20,21,26,30,34,63], menthol [20,67], NSAIDs [2,13,27], and capsaicin [13,20,21,52]. There is a lack of evidence for the use of topical lidocaine and menthol for NP [67]. However, high-concentration (8%) capsaicin generates more pain relief compared to control or low-dose capsaicin [2,63,87] with a reduction in NP after a single 30–60 min application for up to 12 weeks [87]. Capsaicin is an agonist that acts on TRPV1, the heat and capsaicin receptor [87], which may induce desensitization of peripheral nociceptors [2,71]. Various neuromodulating medications, such as fentanyl and ketamine, can be used topically, but evidence of benefit is limited [20,63,87].

#### 3.5.18. Nutrition and Supplementation

There is increasing evidence that a diet rich in antioxidants can promote brain health maintenance [88], best encapsulated by the Mediterranean diet, with well-known antioxidant effects [8,13,88]. The gut microbiome also contributes to the regulation of neurotransmitter signaling relevant to pain modulation by synthesizing and modulating GABA and 5-HT [74]. Obesity is associated with the development of FM [46] and is a negative predictor of anti-CGRP responsiveness in patients with chronic migraine [56]. Additionally, there has been limited evidence regarding the possible advantages of supplementation with vitamin B [8,27], vitamin C [8,59], vitamin E [8,17,89], coenzyme Q10 [53], and fish oil [8,27]. Vitamin B has been shown to be effective in neuropathy and NP. Vitamin B12 increases 5-HT levels and inhibits nociceptive neuronal activity [27]. Ascorbyl palmitate is a fat-soluble ester of vitamin C that has been shown to decrease TN pain and improve the QoL as measured by patient-reported outcome measures (PROMS) [59]. Patients with vitamin E deficiency can present with neuropathy, loss of vibratory and proprioceptive sensation [17,89]. One case study reported marked improvement in symptoms of sensory axonopathy with high-dose vitamin E in the form of D-alpha tocopherol supplementation at 800 IU/day [89]. Vitamin E in humans acts as a free radical scavenger and fat-soluble antioxidant. Vitamin E deficiency has been linked to malabsorption syndromes [89].

Naturally occurring N-acyl ethanolamines have been widely studied in the context of neuroinflammation and NP. Neurons generate oleoylethanolamide (OEA) and palmitoylethanolamide (PEA) in the dorsal roots and many other cell types, even in the absence of external stimuli, and activate peroxisome proliferator-activated receptor-α (PPAR-α), nuclear receptors modulating immune and inflammatory reactions. PEA may modulate the excitability of peripheral nociceptors [15]. It can effectively treat persistent neuroinflammation, including that associated with NP, with no significant adverse effects [2,15].

#### 3.5.19. Visuospatial Techniques

Maladaptive functioning of the M1 has been associated with chronic pain syndromes. Activation of M1 by performing or observing an action reduces chronic pain, which suggests cortical adaptation mechanisms can interact with pain-related circuits [90]. A simple visual illusion can create touch perception [91], and the perception of others’ actions is accompanied by a modulation of the observer’s corticospinal system, resulting in a spatiotemporal muscle activation pattern similar to the agent’s. Improved sensory perception is likely to facilitate motor rehabilitation outcomes. If sensory abilities can be unlocked or enhanced when carefully applied to avoid pain, it may be valuable for patients with sensory impairment [91]. Action Observation Treatment aims to activate the M1 in patients with FM who exhibit pain-related motor impairment [90]. Mirror therapy utilizes a sagitally placed mirror and creates the illusion of moving a hidden limb through the complete mirror inversion of the opposite limb [14]. Mirror therapy and graded motor imagery aim to gradually correct sensorimotor mismatch to integrate vision and kinesthesia and encourage the formation of a body image [35]. Visual information synchronized with somatosensory information seems to produce more potent illusions and a more significant analgesic effect than visual information alone [35]. Body shadows have been used to construct the body image by triggering the perception of movement and can be used to simulate touch without eliciting fear [35]. The role of vision in rehabilitating sensory perception may have been underestimated to date [91]. However, there are limited studies and a low quality of evidence [14,90,91].

#### 3.5.20. Sensory Discrimination Training

Clinical studies reported an association between impaired two-point discrimination, increased pain intensity, and altered cortical representation of the affected body region. Interventions targeting two-point discrimination were designed to normalize cortical representation of the painful area and were reported to improve sensory discrimination and reduce pain [33]. Sensory discrimination training typically involved repetitive exposure to varied tactile stimuli and was associated with changes in cortical representation of the affected region [91]. Desensitization interventions employed light, non-noxious stimuli with gradual increases in stimulus intensity based on patient tolerance and were reported to reduce pain sensitivity [20]. However, improvements were generally slow, adherence rates were low, and evidence for sustained, clinically meaningful long-term effects of therapist-led interventions was limited [33].

#### 3.5.21. Transcranial Magnetic Stimulation (TMS) and Transcranial Direct Current Stimulation (tDCS)

Noninvasive neurostimulation has been proposed to reduce pain perception via an indirect influence on pain modulation areas [14]. The primary M1 is a gateway to deep pain-related networks, including the thalamic nuclei [51]. Transcranial stimulation of the M1 or the dorsolateral prefrontal cortex may inhibit thalamic nociceptive afferents in neural pain pathways, leading to increased release of endogenous opioids and a decrease in the discriminative or affective aspects of pain [14]. Repetitive transcranial magnetic stimulation (rTMS) and transcranial direct current stimulation (tDCS) have shown promising results in inducing corticocortical plasticity with a positive effect on NP processing [14,51]. M1 stimulation can inhibit or excite sensorimotor areas, thus influencing neuroplasticity related to pain and motor improvement [13,32], and are effective treatment modalities for patients with refractory centralized pain and peripheral neuropathy [27,92] and CRPS [92]. Minimally invasive brain stimulation targeting the dorsolateral prefrontal cortex improves acute and chronic pain tolerance [92], and targeting the anterior cingulate cortex has beneficial therapeutic effects in headache, depression, and FM [53]. The safety of tDCS has been well established, and several studies have concluded that tDCS induces only temporary, mild effects, including headache, dizziness, nausea, mild pain, and skin irritation under the electrodes [51]. The clinical effects are modest and short-lasting from a single session. However, repeated sessions may cause more significant and longer lasting effects, opposite to the effect of opioids, which, when used chronically, may increase sensitivity to a noxious stimulus and consequently induce hyperalgesia [51]. Evidence is emerging, and the generalizability of noninvasive neurostimulation remains unclear [59].

#### 3.5.22. Repetitive Peripheral Magnetic Stimulation (rPMS)

A bottom-up approach to increase the flow of proprioceptive signals to the brain to influence neuroplasticity and the mechanisms of pain and motor control could be an alternative to top-down brain stimulation. In one included study, Repetitive peripheral magnetic stimulation (rPMS) was delivered in a single session at a theta-burst frequency of 5 Hz trains of three pulses at 50 Hz during 200 s, 2 s ON/8s OFF, 600 pulses in total. rPMS of muscles is painless and noninvasive and can influence the induction of brain plasticity to reduce pain and improve motricity. The intensity is suprathreshold to trigger muscle contraction and mimic muscle contraction and relaxation to generate afferent proprioceptive information to the brain from the stimulated structures to induce changes in frontoparietal network activity and M1 excitability. rPMS has shown clinical significance of pain reduction, motor improvement, and enhancement of perceptual–cognitive function, which could be explained in terms of long-term potentiation and long-term depression of M1 excitability, which favor central desensitization, all contributing to decreased pain. However, pain was not reduced in all individuals, and rPMS did not lead to improvements across all outcome measures. Only one study met the inclusion criteria for this intervention, limiting synthesis and precluding conclusions regarding efficacy [32].

#### 3.5.23. Alternating Magnetic Fields

An alternative approach to neuronal stimulation is the induction of current using a magnetic field rather than the injection of current via electrodes. Exposure to alternating magnetic fields has been shown to increase intracellular calcium in cultured nerve cells, activate nerve growth factor production in cultured glial cells, and increase neurotrophic messenger ribonucleic acid (mRNA) expression levels in astrocytes, contributing to pain relief. Magnetic field devices have been developed and clinically accepted for transcranial stimulation in the treatment of depression and migraine with aura, and alternating magnetic field devices for FM have shown similar effect sizes as pregabalin. No adverse reactions occurred in either group, suggesting that magnetic stimulation is at least as safe as existing FM treatment methods, with lower pain scores over eight weeks compared with control subjects exposed to a sham device. The findings in this sample support the evaluation of magnetic stimulation in larger-scale studies to determine long term effects [44].

#### 3.5.24. Transcutaneous Electrical Nerve Stimulation (TENS)

Device therapy for FM has principally centered on transcutaneous electrical nerve stimulation (TENS), which delivers pulsed electrical currents across the intact skin surface to stimulate peripheral nerves [44]. TENS stimulates deep sensory afferents that secondarily inhibit nociceptive input, presumably via gate control theory [13,14,27,28,50,53]. Portable, battery-powered TENS devices allow patients to self-administer electrical pulses with varying frequency, amplitude, and duration [44]. However, TENS may not relieve pain adequately [67]. Evidence suggests that TENS reduces pain intensity when administered as a standalone treatment for acute pain [44]. However, the results remain inconclusive for chronic pain [14,21,67].

#### 3.5.25. Photobiomodulation (PBM)

Photobiomodulation (PBM) therapy could be a promising alternative treatment modality strategy in NP management, providing potent and safe analgesia [14,23]. PBM was reported to be associated with increased release of endogenous opioid neuropeptides and neurotransmitters, including β-endorphins, 5-HT, and enkephalins, and reduced release of pronociceptive mediators such as substance P. Reductions in C- and Aδ-fiber activity and modulation of peripheral nerve excitability were also reported, alongside changes in membrane permeability associated with altered cellular activity. Additional reported effects included increased microcirculation, modulation of neurotransmission, enhanced nerve regeneration, increased numbers of proliferating fibroblasts and macrophages, and reductions in inflammatory cytokine levels, with no adverse effects reported [23].

PBM dosing parameters varied substantially across randomized controlled trials. Reported fluences ranged from 1 to 200 J/cm^2^, power outputs from 30 mW to 1 W, and wavelengths from 660 to 980 nm, with substantial variation in exposure time, treatment frequency, and total number of sessions, limiting comparability across studies and contributing to variability in reported outcomes [23].

#### 3.5.26. Bodywork

Manual therapy is a nonpharmacological treatment provided by chiropractors, physical therapists, and osteopaths, among other healthcare professionals, conceptualized as the treatment of dysfunctions in muscles, tendons, ligaments, joints, nerves, skin, and organs performed by the hands of a therapist, covering a variety of refined techniques that aim to mobilize or manipulate the soft tissues of these structures. The primary aim of manual therapy is to increase the range of motion and function, reducing pain [31,55]. Pain modulation may be beyond biomechanics, representing only one possible explanation of this neurobiological effect. The analgesic action of gentle touch therapy (GTT) may be linked to the activation of C-tactile fibers, inducing a limbic response resulting in emotional and hormonal reactions, including increased plasma oxytocin [3,55]. Light skin stroking, able to activate C-tactile units, may activate the posterior insula cortex [3]. One study reported that affective tactile stimulation delivered at 10 cm/s reduced pain and modulated windup compared with control tactile stimulation at 0.3 cm/s. Affective tactile stimulation was rated as more pleasant [37]. Two 45 min GTT interventions 45 days apart have been shown to reduce pain in women with FM but have not demonstrated improvement in the QoL as assessed by the SF-36 [55]. Exteroceptive touch mediated by C-tactile fibers could modulate the efferent activity of the autonomic nervous system [14,37]. Gentle manual therapy does not present contraindications with promising evidence of effectiveness in a range of diverse chronic pain conditions [55]. However, small fiber neuropathy is present in many pain conditions [1,25,37], so in chronic pain, the pain-modulating capacities of C-tactile fibers might be too weak to reduce pain [37].

#### 3.5.27. Acupuncture

Acupuncture is a traditional Chinese medicine therapy that targets specific points along “meridians” that run through the body [8]. Among the many theories postulated to explain the benefits of acupuncture, the included studies reported pain relief due to pain gate theory and the release of endorphins; however, the mechanism underlying pain relief mediated by acupuncture remains inconclusive. Acupuncture may be beneficial in treating different types of NP [27,93], but its efficacy is mainly anecdotal and not supported by high-quality evidence [8,20], with this section being exclusively supported by review studies.

#### 3.5.28. Vagal Nerve Stimulation

Vagal nerve stimulation has been shown to modulate several pathophysiological mechanisms, including decreasing inflammation, reversing activity in brain areas related to pain, and decreasing sympathetic tone. It can be delivered via an implanted device or non-invasively through the skin of the outer ear [38]. Activities that decrease autonomic drive or rebalance autonomic and parasympathetic tone might be considered for people with FM [38]. Patients suffering from psychoaffective components of chronic pain associated with low or high vagal reactivity have been found to benefit from walking and yoga [72], or any preferred exercise [41], showing positive effects on the vagus nerve, claiming parasympathetic resources and thus diverting available attention from rumination and catastrophizing [72].

#### 3.5.29. Vibration

Whole-body vibration [42,43] and localized vibration [3] have been shown to attenuate pain via the activation of mechanoreceptors related to perception, discrimination, and sensory and motor responses [42], with improvement in perceptual balance noted in FM patients [43]. One study reported that vertical and rotational whole-body vibration delivered over 12 weeks (25 Hz; three 45 s bouts per session with 120 s rest) was associated with improvements in FM impact, pain, QoL, vibration sensitivity, functional motor capacity, and balance, with rotational vibration improving a greater number of outcomes than vertical vibration [42]. It was reported that peripheral vibration engaged multiple sensory afferent pathways, including Aβ and C-tactile afferents, with different vibration frequencies associated with differential activation of sensory cortical, cerebellar, and limbic regions, and with both high- and low-frequency localized vibration reported to be associated with oxytocin release and antinociceptive effects [3]. Although the use of vibration has shown positive effects in FM, there is very little evidence for other pain syndromes, and studies are of limited quality. However, vibration is an effective nonpharmacologic intervention and warrants more critical evaluation [3,42].

#### 3.5.30. Bracing and Kinesio Tape

Compression garments may improve proprioception, reduce pain, and prevent recurrent sprains and subluxations in patients with comorbid hypermobility, as seen in Ehlers Danlos syndrome. However, they may not improve fatigue or reduce the need for other therapies and medication. Compression garments are also necessarily tight and may irritate the skin [67]. Kinesio tape may supply a proprioceptive input and stabilize biomechanics where there is joint instability. However, studies assessing its effects have frequently yielded contradictory results [93]. Occlusal equilibration appliances have been trialed for facial pain with some positive results before recurrence [22]. Taping and bracing interventions have been tested across a spectrum of conditions associated with NP. However, the results remain inconclusive [14,22,66].

#### 3.5.31. Exercise

Muscle strength is generally reduced by up to 35% in FM compared with healthy women. This weakness may be explained by pathologic changes in muscle fibers, impaired circulation, disturbances in metabolism, and decreased activity levels associated with pain and kinesiophobia [49]. Physical fitness is associated with improved measures of pain, fear of falling, psychological disorders, and perceived QoL [8,14,88], as assessed by instruments explicitly designed to capture overall QoL, health-related QoL, or life impact, primarily the Fibromyalgia Impact Questionnaire (FIQ) and Short Form Health Survey (SF-36) [36,43,72]. Aerobic and resistance exercise are effective in increasing strength, improving balance, and enhancing exercise tolerance [13,43,46], and in reducing pain in FM [8,39,40] and chronic NP [13,14]. The beneficial effects of exercise on central pain modulation may, in part, be mediated by serotonergic mechanisms, including alterations in 5-HT transporter expression, increases in central 5-HT availability, and enhanced activity of endogenous opioid pathways [14]. Three studies reported outcomes associated with exergaming interventions. Improvements in heart rate variability were reported following two hours of exergaming per week over 24 weeks [41]. Increases in lower limb strength were reported following either three 1 h sessions per week for 20 sessions or two 1 h sessions per week over 24 weeks [39,40]. Reductions in hyperalgesia were reported after three 1 h sessions per week for 20 sessions [39]. Improvements in cardiovascular fitness were reported following two 1 h sessions per week over 24 weeks [40]. Home exercises, including exergaming [39,40,41], are cost effective and may increase compliance, which is a well-documented issue [8,40]. Exercise has been shown to alter 5-HT transporter expression, increase 5-HT levels, increase opioid levels in central inhibitory pathways, and facilitate inherent inhibitory systems to modulate pain [14]. Physical exercise is one of the nonpharmacological therapies with the highest level of evidence to manage FM symptoms [41]. The addition of proprioception exercise to FM rehabilitation (40–60 min, 3 times per week for 12 weeks) has been shown to significantly improve balance, mobility, pain management, fatigue, and muscle weakness, resulting in reduced falls and improved QoL [43].

#### 3.5.32. Psychological Support

Psychiatric comorbidities are common in chronic pain conditions [6,12,30]. Psychosocial factors, including pain catastrophizing and illness perceptions, are significant mediators of reported pain intensity, suggesting altered somatosensory processing, and central integration of these phenomena [6,8,27,42]. Cognitive changes accompanying chronic pain often involve disproportionate attentional selection of pain at the expense of the remaining sensory inputs [72]. Hypervigilance to pain is associated with kinesiophobia [46], so pain-related fear tends to predict functional disability beyond pain intensity [5]. Psychological counseling and support may help people identify a more effective strategy to manage pain characterized by catastrophic thinking and hypervigilance [14,20,72]. Cognitive behavioral therapy is the primary psychologically based treatment for patients with centralized pain [4,6,8,20,34,44,53,94]. Distraction from negative thoughts may interrupt the perception of nociceptive stimulation by detaining the ruminative or catastrophizing thoughts associated with pain from the physiological resources supporting them [72]. A high sense of coherence allows patients to better cope with stressors and represents a health promoting factor associated with increased wellbeing and reduced incidence of depression [88]. Referral to mental health specialists should be suggested to all patients in whom mood disorders are known or suspected. Tools, including the Beck Depression Inventory (BDI), may be helpful for screening [5,8]. However, patients must understand that such a referral is an integral part of their treatment, not a confirmation that their pain is psychosomatic [8,9].

#### 3.5.33. Multidisciplinary Approach

An individualized and multidisciplinary healthcare team appears to best cater for the management of NP and CS [5,6,20,21,22,31,43,44,55,63,94]. The development of personalized pain biomarkers will be central to accurate diagnosis, tracking prognosis, and future therapeutic development [2,56]. However, many treatments currently in practice lack robust evidence, as recommended by the guidelines [8,29,32]. Evidence-based outcome measures are needed to assess the impact of new treatment interventions and treatment interactions [45].

Diagnosing pain without apparent tissue damage is complicated [1,47]. The disparity between signs and symptoms often results in patients being considered malingering, hysterical, or psychosomatic [27,47]. Pain sensitivity and treatment response are characterized by multiple variables, including differences in nociceptive sensitivity, psychological factors, sociocultural factors, environmental influences, and genetic variation [24]. Few existing treatments effectively reduce neurogenic pain in the long term [25,38]. Patients often erroneously believe that medical therapy can completely alleviate their symptoms, that health professionals do not understand their condition, and that their health outcomes are affected by this lack of knowledge [9]. Healthcare practitioners need to communicate that the goal of medical therapy is not to eradicate pain but to decrease symptoms to an acceptable level and provide coping strategies [22]. To develop a treatment that meets the patient’s needs and obtain long-term adhesion, it is essential to understand the patient’s perspectives, including their expectations, their experience of interventions, and their lifestyle [26,94]. Pain education is effective, and clinician empathy promotes improved outcomes [20,94]. Eliminating pain may not be possible, but improving the QoL for the patients is nearly always achievable [9].

**Table 1:** Summary of interventions and observations.

Author	Study Numbers	Condition	Intervention/ Observation	Result
Allen Demers et al., 2021 [32]	5 female, 3 male, mean age 55.7 ± 9.7 (35–65)	CRPS	Repetitive peripheral magnetic stimulation	rPMS reduced pain, improved proprioception and range of motion.
Alqahtani and Parveen, 2023 [93]	Review	Masticatory myofascial pain syndrome	Kinesio taping	Reduction in edema and discomfort. May be adjunctive therapeutic tool but not a valid independent treatment option.
Anand et al., 2019 [87]	8 female, 8 male, mean age 64 (45–79)	Peripheral neuropathy	Capsaicin patch	Capsaicin 8% patch provides significant pain relief in peripheral neuropathy, and may lead to regeneration and restoration of sensory nerve fibers, i.e., disease modification.
Andersen et al., 2022 [57]	62 female, 53 male	Trigeminal neuralgia	Microvascular Decompression	Significant association between excellent surgical outcome and male sex and neurovascular contact with morphological changes.
Antunes et al., 2022 [94]	12	Fibromyalgia	Interdisciplinary program	Interdisciplinary educational programme emphasizes self-management strategies.
Araya et al., 2020 [63]	Review	Trigeminal neuralgia	Pharmacological and surgical interventions	Risks and benefits for pharmacological treatment options with varying individual indications and success rates.
Baksh et al., 2021 [50]	Review	Dry eye, migraine	Nil	Commonly comorbid conditions with possible shared pathophysiology. Understanding relationship between comorbid conditions may improve treatment strategies.
Barbanti et al., 2022 [56]	675 female, 189 male, mean age 47.8 ± 11.5	Migraine	Anti-CGRP monoclonal antibodies	Peripheral or central sensitization symptoms may help predict response to anti-CGRP monoclonal antibodies.
Bartindale et al., 2018 [81]	Review	Trigeminal neuralgia, hemifacial spasm	Microvascular decompression	TN showed hearing loss of 5.58%, hemifacial spasm showed hearing loss of 8.25% following MVD
Bartindale et al., 2020 [83]	131 females, 52 males, mean age 58.52	Trigeminal neuralgia, hemifacial spasm, glossopharyngeal neuralgia, vagal palsy, tinnitus	Microvascular decompression	Complications in 17.7%. 4.17% with permanent hearing loss, 6.77% with transient hearing loss, 5.21% with tinnitus, 5.70% with vertigo, 0.52% with HFS.
Benistan et al., 2023 [67]	61 female, 6 male, mean age 33.1 (16–60)	Non-vascular Ehler’s Danlos Syndrome	Compression garments	Compression garments effective for reducing pain for the most painful joint, other joints and for measures of NP.
Berryman et al., 2021 [38]	29 female, mean age 45.63 ± 14.41	Fibromyalgia	Assessment and treatment	Prepulse faciliation was enhanced in the FM group suggests alteration in information processing linked to autonomic drive.
Bravo et al., 2019 [36]	Review	Fibromyalgia	Exercise and body awareness	Positive results in favor of movement and body awareness therapies as adjunct treatment to usual care.
Byrom et al., 2023 [45]	244, mean age 37.8 (16–69)	Fibromyalgia	Nil	Data obtained from social media offers insight into living with chronic pain which can influence the development of patient reported outcome measures.
Casale et al., 2022 [3]	Review	Nociception	Localized vibration	Aβ-fibers activation most effective antinociception activated at frequencies between 100 and 250 Hz.
Cattaneo et al., 2020 [88]	3131 female, 1555 male, mean age 53.2 (45–60)	Neurological and neuropsychiatric disease	Lifestyle (diet, exercise, sleep)	Female gender, poor sleep quality and low sense of coherence predicted the onset of a new diagnosis. Healthy lifestyle associated with improved sleep quality and mental health.
Cetera et al., 2023 [8]	Review	Endometriosis	Assessment of pain contributors	Pain contributors include inflammation, peripheral sensitization, CS, myofascial disorders and psychopathological conditions.
Chan et al., 2021 [89]	Case Study	Sensory axonopathy	Vitamin E	Vitamin E deficiency can be a cause of sensory axonopathy.
Chang et al., 2020 [4]	Review	CRPS	Nomenclature	The authors argue for reclassification of CRPS as an functional neurological disorder.
Chiaramonte et al., 2019 [43]	84 female, 42 proprioceptive exercise, 42 control, (20–40)	Fibromyalgia	Traditional exercise plus proprioceptive training	Combination of traditional exercises and proprioceptive training reduced pain and fatigue and increased muscular performance in FM.
Choi et al., 2022 [7]	233 female, 167 male, mean age 61.59 ± 11.94	Peripheral neuropathic pain	Assessment of PainDETECT questionnaire (Korean)	Korean PD-Q effective in subgrouping of peripheral NP by sensory symptom profile, aiding effective personalized treatment decisions.
Cigarán-Méndez et al., 2022 [46]	126 female, mean age 52.0 ± 10.7	Fibromyalgia	Correlation of handgrip and timed up and go test with physical function and fear avoidance	Hand grip weakness associated with greater sensitization, pain intensity, poorer QoL, functional ability and sleep quality. Larger TUG scores associated with greater sensitization, poorer QoL functional ability, pain catastrophism and kinesiophobia.
Coats et al., 2020 [16]	28 female, 14 male, mean age 60.64 ± 15.67 (24–84)	Trigeminal neuralgia	Assessment of sensorimotor and cognitive tasks	TN group taking antiepileptic drugs performed worse than controls on sensorimotor tracking and aiming tasks and across all cognitive measures.
Coggins et al., 2023 [33]	Proof of concept study aiming for 20 participants	CRPS	Sensory Training Device	The use of sensory training devices in CRPS may improve tactile acuity and reduce perceived pain.
Cruz Salcedo et al., 2020 [29]	Case study	CRPS	Snake bite as contributing factor	The study aims to increase awareness of CRPS clinical recognition.
Davydov et al., 2021 [72]	110 female with FM, mean age 52.17, 60 HC mean age 49.32	Fibromyalgia	Personalized behavioral intervention	Increased externally oriented thinking associated with lower depressive symptoms and reduced need for medication in FM.
de Carvalho et al., 2021 [39]	35 female FM patients, 16 Wii, mean age 55.64, 19 CG mean age 47.70	Fibromyalgia	Exergaming	Exergaming produces a decrease in tender point count in women with FM.
Doğan et al., 2021 [95]	Case study (2)	Neuropathic pain	Pregabalin	The authors suggest pregabalin use associated with the development of vision loss due to central serous chorioretinopathy.
Dudulwar et al., 2022 [48]	Case study	Trigeminal neuralgia	Lipoma as contributing factor	Cerebellopontine angle lipoma presented as TN. Excision associated with hearing loss and facial palsy.
Ebrahimiadib et al., 2020 [6]	Review	Ocular neuropathic pain	Assessment and treatment	Multidisciplinary approach, collaborating ophthalmologists and pain specialists, can be effective in reducing hyperalgesia and allodynia.
Eskandar et al., 2023 [19]	Review	Trigeminal neuralgia	Radiofrequency ablation	Radiofrequency ablation provides pain relief and improved QoL and is a safe and effective alternative to other surgical procedures.
Fidanza et al., 2021 [37]	24 female, 18 male, mean age 28.07 ± 8.8 (19–53)	Central sensitization	Affective and discriminative touch	Affective touch modulated CS. Discriminative touch modulated relationship between body awareness and pain.
Finco et al., 2020 [2]	Review	Chronic musculoskeletal pain	Assessment and treatment	Early detection and tailored, mechanism treatment required to restrain the reinforcement of pronociceptive remodeling.
Finnerup et al., 2021 [1]	Review	Neuropathic pain	Mechanisms	Understanding of pathophysiology has increased but not translated into improvement in treatment.
Fitzgerald et al., 2022 [47]	1741 female 2724 male, mean age 44.5 ± 14.0	Fibromyalgia	Spondyloarthritis as contributing factor	The presence of FM inflates disease severity in individuals with enthesitis.
Garcia et al., 2023 [76]	Review	Nociplastic pain	Spinal cord stimulation	Spinal cord stimulation has proven its efficacy in refractory and difficult-to-treat pain syndromes.
Gentile et al., 2022 [90]	22 FM patients, 19 female, 3 male 50.45 ± 10.67 20 HC 13 female, 7 male 46.30 ± 11.48	Fibromyalgia	Movement observation	Movement observation activates motor networks. Activation of the M1 is known to induce an analgesic effect in patients with chronic pain.
Hagenberg et al., 2022 [91]	Review	Sensory impairment	Mirror therapy	Referral of sensation elicited in mirror therapy indicates potential benefits for sensory rehabilitation.
Hanna et al., 2022 [23]	PBM 15 female, 3 male, mean age 58.00 ± 10.39, MED 10 female 56.80 ± 10.84	Oral neuropathic pain	Photobiomodulation	Safety and efficacy of laser-PBM demonstrated in modulating NP intensity, improving functionally and QoL indicating a possible therapeutic option for oral NP.
He and Kim, 2022 [20]	Review	Allodynia	Assessment and treatment	Allodynia is multifactorial. Interprofessional healthcare team offers best management.
Henshaw et al., 2021 [70]	Review	Inflammation	Cannabidiol, 9-tetrahydrocannabinol	CBD + THC combination exert an antiinflammatory effect whereas THC alone does not reduce proinflammatory or increase antiinflammatory cytokines.
Hirakata et al., 2018 [12]	23,255 female, 22,076 male, mean age 66.8 ± 13.9	Neuropathic pain and Fibromyalgia	Pregabalin	In Japan, number of patients being prescribed pregabalin increased but doses decreased, possibly due to adverse effects.
Hirakawa et al., 2020 [35]	Case Study	CRPS	Body shadow intervention	Body shadows can alleviate pain by creating potent body illusions that simulate touch without eliciting fear.
Jain and Moorthy, 2022 [10]	Review	Rheumatoid arthritis, Osteoarthritis and Fibromyalgia	Cannabinoids	Knowledge still lacking about the efficacy, dosing and drug interactions of cannabinoids.
Johnston-Devin et al., 2022 [9]	14 female, 3 male, mean age 44 (22–65)	CRPS	Patient interview	Patients perceive that health professionals do not know enough about CRPS, leading to poor treatment decisions and health outcomes.
Kannan et al., 2022 [14]	Review	Neuropathic pain	Physiotherapy	Evidence for non-invasive neurostimulation for spinal cord injury and phantom limb pain, mirror therapy for phantom limb pain, acupuncture for NP secondary to stroke, and TENS and exercises for multiple sclerosis.
Kayani et al., 2022 [58]	Review	Trigeminal neuralgia	Botulinum toxin	Clinical efficacy for botulinum for TN, with improvement in pain frequency and intensity. Concern regarding drug interaction.
Khijmatgar et al., 2022 [59]	4 female, 7 male, mean age 55.36 ± 10.67	Trigeminal neuralgia	Ascorbyl palmitate	Ascorbyl palmitate prevents frequent exacerbation of pain and improves patient QoL in TN.
Kocamaz and Karadag, 2019 [65]	Case report	Epilepsy	Topiramate	Acute myopia, diplopia, and photosensitivity induced by topiramate prescribed for epilepsy.
Kuvatanasuchati and Leowsrisook, 2021 [22]	Case report	Facial pan	Occlusal equilibration appliance	Occlusal equilibration appliance reduced pain in TN, offering alternative to more invasive and expensive treatments.
Labau et al., 2022 [25]	Review	Neuropathic pain	Human induced-pluripotent stem cells for study	Human induced-pluripotent stem cells are an alternative to study patient-specific diseases.
Labetoulle et al., 2019 [13]	Review	Dry eye disease and Neurotrophic keratopathy	Assessment and treatment	Therapies targeting nerve regeneration may treat NK and counteract DED-perpetuating factors, e.g., hyperosmolarity, tear secretion and inflammation.
Lagomarsino et al., 2021 [74]	Review	Chronic pain	Microbial-sensory neuron crosstalk as contributing factor	Studies to examine microbial-sensory neuron crosstalk in nociception may lead to new therapies.
Lee et al., 2021 [85]	54 female, 34 male, mean age 56.9 (29–82)	Trigeminal neuralgia	Microvascular decompression and partial sensory rhizotomy	MVD cure rate 68.8%. Partial sensory RHZ cure rate 54.5%. Partial sensory RHZ considered when no significant vascular compressive lesion.
Li et al., 2023 [17]	Review	Neuropathic pain	Excessive Iron Accumulation as a contributor	Elevated intracellular ROS in spinal cord dorsal neurons induce neuronal ferroptosis pathway and participate in the process of NP.
Lind et al., 2022 [15]	7 female, 14 male with NP, mean age 69.7 ± 9.1, 19 male, 8 female 71.6 ± 7.5	Chronic idiopathic axonal neuropathy	Levels of bioactive endogenous lipids as contributing factor	Alterations of 2-arachidonoylglycerol levels in polyneuropathy indicate that it could play a role.
Liu et al., 2022 [69]	Case report	Trigeminal neuralgia	Cavernoma as contributing factor	Cavernoma involving cranial nerves is rare.
Maldonado and De Jesus, 2023 [21]	Review	Hyperesthesia	Assessment and treatment	Mainstay of treatment is symptomatic relief via pharmacological, non-pharmacological, and interventional therapies.
Malfitano et al., 2021 [92]	Case study	Central post stroke pain	Repetitive Transcranial Magnetic Stimulation	rTMS treatment was associated with decreased pain and M1 excitability changes.
Mastronardi et al., 2020 [84]	49 female, 22 male, mean age 59.8 ± 5.4 (40–84)	Trigeminal neuralgia	Microvascular decompression	88.7% had good pain outcome. 9.9% had recurrence of pain. Sensorineural hearing loss and other complications possible.
Maurya et al., 2019 [80]	24 female, 27 male	Trigeminal neuralgia	Assessment and treatment	Neurovascular contact between trigeminal nerve and vessel was seen in 41 (80.4%) cases and 17 (28.3%) controls.
McDonagh et al., 2020 [52]	Review	Chronic pain	Nonopioid pharmacologic agents	Small improvement seen with SNRI for NP, FM, osteoarthritis, and low back pain; gabapentanoids for NP and FM; oxcarbazepine for NP; and NSAIDs for osteoarthritis and inflammatory arthritis.
Mingorance et al., 2021 [42]	54 female, 6 male, mean age 52.4 ± 8.4 (35–65)	Fibromyalgia	Whole body vibration	Significant but not lasting improvement with rotational WBV and not vertical WBV.
Morgalla and Domay, 2022 [77]	9 female, 5 male, mean age 58.4	Neuropathic pain	Spinal cord stimulation	Some normalization of sensory testing and fiber function seen with use of burst or tonic stimulation. Burst stimulation superior.
Moshirfar et al., 2023 [27]	Review	Ocular neuropathic pain	Assessment and treatment	Best approach is with interprofessional team.
Muller et al., 2019 [71]	Review	Neuropathic pain	Cannabinoids	Cannabinoid ligands exert numerous physiopathological functions by modulating TRP channels, influencing pain perception
Oka et al., 2020 [44]	39 female, 1 male, mean age 52.6 ± 14.1	Fibromyalgia	Magnetic field device	Reduction in pain scores comparable to pregabalin and duloxetine. No adverse reactions.
Pak et al., 2022 [60]	15 female, 10 male, mean age 63 ± 10.4	Trigeminal neuralgia	Endoscopic microvascular decompression	Endoscopic MVD improves intraoperative visualization but similar surgical risks and short-term clinical outcomes as microscopic MVD.
Patel et al., 2020 [30]	Case study	CRPS	Axillary lipoma as contributing factor	CRPS a possible postsurgical complication.
Rhee et al., 2019 [28]	Case study	CRPS	Dental treatment of CRPS patient	Ketamine used for sedation provided pain reduction.
Sabatschus et al., 2022 [26]	Review	Peripheral neuropathic pain	Topical lidocaine	Lidocaine 700 mg medicated plaster has more favorable benefit–risk balance than pregabalin.
Sachau et al., 2019 [24]	121 female, 107 male, mean age 51.3 ± 18.7	Neuropathic pain	SIGMAR1 gene mutation as contributing factor	Significant modulation of somatosensory function in NP patients by genetic variants in SIGMAR1.
Salgado et al., 2022 [55]	64 female, 32 GTT mean age 53.7 ± 9.6, 32 control mean age 53.2 ± 8.2	Fibromyalgia	Gentle Touch Therapy	Lower pain score was observed in GTT group without altering the QoL. Serum BDNF at baseline predicted impact on pain measures.
Schoenen and Coppola, 2018 [53]	Review	Migraine	Noninvasive neurostimulation—external trigeminal nerve stimulation	eTNS viable alternative to pharmacological antimigraine strategies chiefly exert its action by modulating perigenual anterior cingulate cortex.
Schranz et al., 2020 [31]	Case study	CRPS	Counterstrain	Counterstain showed lasting resolution of symptoms and improved proprioception and temperature discrimination in affected limb.
Shi et al., 2022 [66]	Review	Neurovascular compression	Treatment and assessment	In HFS, TN, glossopharyngeal neuralgia, and paroxysmal vestibular syndrome the etiology of neurovascular compression should be considered.
Skinner and Kumar, 2021 [75]	Case study	Atypical occipital neuralgia	Ultrasound-guided occipital nerve block	Ultrasound-guided occipital nerve block is low-risk therapeutic intervention for occipital neuralgia and atypical facial pain.
Smulders et al., 2021 [54]	Case study	Trigeminal neuralgia	Radiofrequency treatment of the trigeminal ganglion	Trochlear nerve palsy resulting in diplopia is a rare, generally transient complication of percutaneous radiofrequency treatment of the trigeminal ganglion.
Su et al., 2023 [34]	Case study	CRPS	Subcutaneous lidocaine injection	Local subcutaneous injection of 2% lidocaine effective in relieving complex local pain.
Sun et al., 2020 [79]	12 female, 8 male, mean age 55 (48–71)	Trigeminal neuralgia	Endoscopic microvascular decompression	Compared with traditional microscopy, endoscopy has advantages that can improve clinical outcomes.
Tavares et al., 2018 [51]	Proof of concept study	Nociplastic knee pain	Transcranial Direct Current Stimulation	tDCS of M1 may decrease pain where there is defective pain modulation.
Tavares et al., 2020 [5]	88 female, 16 male, mean age 73.9 ± 8.01	Knee osteoarthritis	Assessment and treatment	Central and peripheral sensitization and psychological factors influence the experience of chronic pain due to knee osteoarthritis.
Ungar et al., 2018 [78]	28 female, 26 male mean age 59 (25–80)	Auditory nerve neurovascular contact	Assessment and treatment	Concludes no association between vestibulochochlear neurovascular contact and unilateral sensorineural hearing loss.
Vaghela et al., 2023 [49]	Case study	Neuropathic pain, Fibromyalgia, and Chronic musculoskeletal pain	Polyarthropathy	Duloxetine identified as potential cause of heartburn, one of its rare adverse events.
Villafaina et al., 2019 [40]	22 female exergaming, mean age 54.27 ± 9.29, 15 female control mean age 53.44 ± 9.47	Fibromyalgia	Exergaming	Exergaming improved lower body strength and cardiorespiratory fitness in women with FM. Exergames must be performed regularly to maintain strength benefits.
Villafaina et al., 2020 [41]	28 female exergaming, mean age 54.04 ± 9.96, 27 female control, mean age 53.41 ± 9.92	Fibromyalgia	Exergaming	24 weeks of exergame intervention improved autonomic control in patients with fibromyalgia but with no impact on HRV.
Wang et al., 2023 [62]	18 female, 21 male MVD, mean age 62.87 ± 13.17, 11 female, 11 male RHZ, mean age 62.45 ± 12.94	Glossopharyngeal neuralgia	Microvascular decompression or rhizotomy	MVD or RHZ effective in treating glossopharyngeal neuralgia.
Wang et al., 2019 [82]	14 female, 9 male	Trigeminal neuralgia	Microvascular decompression	MVD an effective, reliable, and safe neurosurgery for treatment of TN compressed by the vertebrobasilar artery.
Wasim et al., 2022 [61]	38 female, 19 male (27–90)	Trigeminal neuralgia	Radiofrequency ablation	Radiofrequency ablation effective treatment for TN with lower recurrence rate but higher sensory deficits compared to MVD.
Yılmaz et al., 2020 [68]	Case report	Neuropathic pain	Pregabalin	Pregabalin should be administered with caution due to potential side effects including hearing loss.
Yousaf et al., 2022 [73]	Review	Neurological disorders	Cannabidiol	CBD supported for treating neurological disorders through addressing microglia-mediated neuroinflammation.
Zhi et al., 2023 [86]	62 female, 25 male mean age 64.76 ± 11.65 (39–86)	Trigeminal neuralgia	Percutaneous balloon compression	Percutaneous balloon compression under conscious sedation local anesthesia is an effective minimally invasive procedure for the treatment of primary TN.

Abbreviations: CRPS, Complex Regional Pain Syndrome; rPMS, repetitive Peripheral Magnetic Stimulation; CGRP, Calcitonin Gene Related Peptide; TN, Trigeminal Neuralgia; NP, neuropathic pain; Hz, Hertz; CS, Central Sensitization; PD-Q, PainDETECT questionnaire; TUG, Timed Up and Go test; QoL, Quality of Life; M1, Motor Cortex; PBM, Photobiomodulation; CBD, Cannabidiol; THC, Tetrahydrocannabinol; TENS, Transcutaneous Electrical Nerve Stimulation; DED, Dry Eye Disease; NK, Neurotrophic Keratopathy; MVD, Microvascular Decompression; RHZ, Rhizotomy; ROS, Reactive Oxygen Species; rTMS, Repetitive Transcranial Magnetic Stimulation; SNRI, Serotonin Noradrenaline Reuptake Inhibitor; NSAIDs, Nonsteroidal Antiinflammatory Drugs; WBV, Whole Body Vibration; TRP, Trans Receptor Potential; GTT, Gentle Touch Therapy; BDNF, Brain Derived Neurotrophic Factor; eTNS, external Trigeminal Nerve Stimulation; HRV, Heart Rate Variability.

## 4. Conclusions

The aims of this systematic review were to explore alterations in multisensory processing associated with chronic pain conditions and to review current pharmacological and nonpharmacological treatment strategies. The systematic review shows that there are many overlaps between conditions of NP and CS, with high rates of comorbidity and high personal and societal costs, with no one treatment offering complete symptom resolution. The persistence of symptoms and refractoriness to treatment could be due to central changes that are not sufficiently influenced by conventional approaches. High rates of overdoses on pain medication in patients with chronic pain need new approaches to effective and harmless pain control. However, nonpharmacological approaches in pain management have had little high-quality research and show varying efficacy. A lack of biomarkers and personalized pain approaches has prevented alternative forms of treatment from achieving adequate efficacy. Cranial neurovascular compromise is increasingly recognized as a source of local NP as neuroimaging becomes more sophisticated. Monoclonal antibodies are effective in a subset of migraine sufferers. The acknowledgement of neuroplasticity of the nervous system has encouraged the use of cortical stimulation, which is an emerging and promising field. Visuospatial techniques and Vitamin B12, Vitamin E, and PEA supplementation may have some effectiveness without recorded adverse effects; however, studies are limited. Exercise has proven to be effective in pain management; however, compliance is an issue, especially when there is pain catastrophizing or kinesiophobia. A multidisciplinary approach to diagnosis and pharmacological, somatic, and psychological treatment is agreed to have the best outcomes. The patient’s experience must be voiced and acknowledged to achieve genuine patient-centered care. Including patients as a healthcare team member is recommended to enable control and self-management of their pain. A tailored, patient-centered approach should restore QoL by considering the multiple mechanisms that modify the nervous system. Interventions that integrate somatic, physical, and emotional factors should be considered when developing clinical programs, although the effects of most therapies are modest. Sometimes, response to therapy can provide clues to pathogenesis. Future research is needed to elucidate the pathogenesis and maintenance of NP and CS syndromes and to identify the most effective treatment strategies.

### Limitations

Despite the structured search strategy, the findings must be interpreted in the context of several limitations. The methodological approach used here may limit comparability to prior studies. The inclusion of reviews provides broad context of the current state of research, but may introduce author bias, limiting the ability to judge the quality of evidence. No statistical syntheses were conducted. Several medical conditions comorbid with NP and CS were excluded, which may also reduce generalizability to other chronic pain populations with one or more comorbid conditions. The findings of all conditions were assessed concurrently, with an assumption of a central origin.

**Going Forward**: Patients presenting with physical symptoms of pain for which no disease cause can be found are primarily female and prevalent across all medical disciplines. Treatment protocols to date, although numerous, have been mostly ineffective in the case of nociplastic pain. Advances in medical imaging may improve diagnosis, though a definitive biomarker has yet to be identified. The recommendation from this systematic review would be for collaborative, multidisciplinary, patient-centered care, with nonpharmacological therapy as the first-line therapy. Physical exercise is one of the nonpharmacological therapies with the highest level of evidence, and the addition of proprioceptive and vestibular exercise has been shown to improve QoL. However, it must be introduced within the individual metabolic capacity to encourage compliance. Cortical, vagal, and peripheral magnetic or photostimulation to evoke plasticity in the neuraxis is an emerging and promising therapy with only mild reported adverse effects and warrants further investigation. Validating individual health experiences and including patients in their treatment plan allows them control and offers the ability to self-manage their pain.

## Data Availability

No new data were created or analyzed in this study. Data sharing is not applicable to this article.

## Supplementary Materials

The following supporting information can be downloaded at https://www.mdpi.com/article/10.3390/ijms27031231/s1.
